# Improved Alere Determine Lipoarabinomannan Antigen Detection Test for the Diagnosis of Human and Bovine Tuberculosis by Manipulating Urine and Milk

**DOI:** 10.1038/s41598-019-54537-9

**Published:** 2019-11-29

**Authors:** Juan Ignacio García, Holden V. Kelley, Johanna Meléndez, Rosa Alejandra Alvarez de León, Alejandra Castillo, Sabeen Sidiki, Kizil A. Yusoof, Elizabete Nunes, Cesar López Téllez, Carlos Rodolfo Mejía-Villatoro, Janet Ikeda, Alberto L. García-Basteiro, Shu-Hua Wang, Jordi B. Torrelles

**Affiliations:** 1 PhD Programme in Methodology of Biomedical Research and Public Health, Department of Paediatrics, Obstetrics and Gynaecology, Preventive Medicine, and Public Health, Universitat Autònoma de Barcelona, Barcelona, Spain; 2 Centro de Investigação em Saude de Manhiça (CISM), Maputo, Mozambique; 3 Texas Biomedical Research Institute, San Antonio, TX 78256 USA; 4 Unidad de Atención Integral del VIH e Infecciones Crónicas del Hospital Roosevelt “Dr. Carlos Rodolfo Mejía Villatoro”, Ciudad de Guatemala, Guatemala, Guatemala; 5 Sección de Microbiología, Departamento de Laboratorios Clínicos, Hospital Roosevelt, Ciudad de Guatemala, Guatemala, Guatemala; 6 Clinica de Atención Integral Dr. Isaac Cohen Alcahe, Hospital de Especialidad Dr. Robles, Quetzaltenango, Guatemala; 7 Department of Microbial Infection and Immunity, College of Medicine (COM), The Ohio State University (OSU), Columbus, OH 43210 USA; 8 Hospital Central de Maputo, Division of Pulmonology, Maputo, Mozambique; 9 Investigación, Desarrollo, y Educación Integral, Quetzaltenango, Guatemala; 10 ISGlobal, Hospital Clínic - Universitat de Barcelona, Barcelona, Spain; 11 Amsterdam Institute for Global Health and Development (AIGHD), Amsterdam, The Netherlands; 12 Department of Internal Medicine, Division of Infectious Diseases, COM, OSU, Columbus, USA

**Keywords:** Infectious-disease diagnostics, Laboratory techniques and procedures

## Abstract

Tuberculosis (TB) disease still kills 1-person every 21-seconds. Few TB diagnostic tests are considered truly appropriate for point of care settings. The WHO-endorsed immunodiagnostic Alere Determine Lipoarabinomannan Ag-test (LAM-test) detects *Mycobacterium tuberculosis* complex LAM in urine, and its use is recommended for TB diagnosis among HIV co-infected individuals with low CD4 T-cell counts. Here we found that a simple 15-minute enzymatic treatment at room temperature of LAM-spiked urine with α-mannosidase (for human TB), and LAM-spiked milk with combined lactase and caseinase (for bovine TB), enhanced 10-fold the detection levels of the LAM-test and thus, improved the detection of LAM by the LAM-test in urine and milk that otherwise could be missed in the field. Future separate clinical research studies specifically designed to address the potential of these findings are required.

## Introduction

Tuberculosis (TB) is the leading cause of death by a single infectious disease^1^. The *Mycobacterium tuberculosis* (*M.tb*) complex cell wall has many cell wall components that are being evaluated for diagnosis purposes. One of them is the lipoglycan so called mannose-capped lipoarabinomannan (LAM)^2–4^, which is found in urine of active TB patients^2–8^. To date, the only WHO supported TB point-of-care (POC) test is the Alere Determine Lipoarabinomannan (LAM) Ag test (LAM-test)^9^. This test is based on polyclonal antibodies of unknown specificity recognizing LAM in urine, as an indicator of TB disease^7,10^. The LAM-test best performance is in HIV-positive individuals with CD4 T cells counts below 50/mm^3^ of blood (52% sensitivity/98% specificity)^7,10,11^.

**Figure 1:**
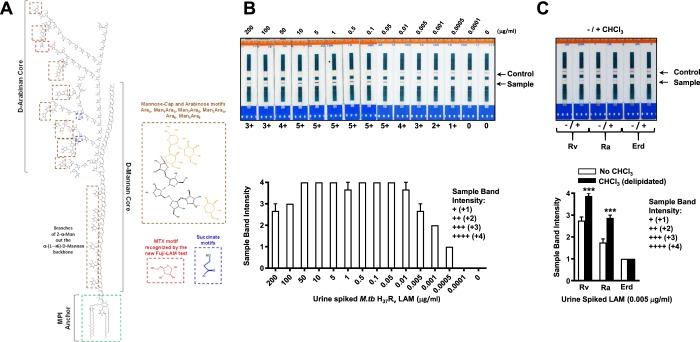
(**A**) Structure of mannose-capped lipoarabinomannan (ManLAM) present in all *M.tb* complex strains. ManLAM (depicted here as LAM) is a heterogeneous molecule comprised of a GPI-anchor, which can contain from 1–4 fatty acids, an α-(1 → 6) mannan core with multiple branches of a single mannose, an α-(1 → 5) arabinan core with multiple branches of different length at the C3 position of some arabinoses. The non-reducing end of some of these arabinan branches are decorated with 2-α-mono-, di- and tri-mannosaccharide caps. A 5-methyl-thio-xylose (MTX) is present per LAM molecule, being the epitope recognized by the new FujiLAM test. LAM also contains succinate motifs, which biological function is still unclear but participate in determining the spatial conformation of LAM. (**B**) Alere Determine LAM Ag test (LAM-test) performed in *M.tb* H_37_R_v_ LAM spiked urine determining that the lowest amount that this test can detect LAM in urine is 0.0005 μg/ml of urine (500 pg). (**C**) A quick delipidation step for LAM spiked urine using chloroform (CHCl_3_) improves the detection of LAM by the LAM-test. Student’s *t* test, treatment *vs*. non-treatment, n = 3–8, using LAM spiked urine from different human donors; ***p < 0.0005.

To improve the sensitivity of this test, we closely looked at the structure of the *M.tb* complex mannose-capped LAM or ManLAM (reviewed in^4^), ManLAM (from here on called LAM) is composed of a GPI-anchor, arabinan and mannan domains, and mannose caps^4^. The number of caps, length and branching of the arabinan and mannan domains, number of succinates, and the number and nature of fatty acids in the GPI-anchor makes this an extremely heterogeneous molecule^4^. A unique methylated thio-xylofuranose (MTX) is attached to a single mannose-cap of LAM^4^, being described in susceptible and drug resistant *M.tb* strains (Fig. 1A)^4^. MTX detection is the basis for the new (Foundation for Innovative New Diagnostics (FIND) supported POC Fujifilm/SILVAMP TB-LAM (FujiLAM) test, being tested in field diagnostic validation trials^12–14^.

Based on the high LAM structural complexity^4^, and the low sensitivity of the LAM-test^7,10,11^, we rationalized that the complex LAM molecule could be associated with other molecules (phospholipids, immunoglobulins, creatinine, etc.) in urine and/or milk that could made it less likely to be recognized by antibodies^15,16^. Thus, we biochemically treated LAM-spiked urine or milk to improve the LAM-test performance for the diagnosis of human and bovine TB. Out of several treatments tested (organic solvent delipidation, Proteinase-K, non-specific esterase, phospholipase, phosphatase, urease, creatinase, α-mannosidase, caseinase, and/or lactase treatments), our results showed that α-mannosidase treatment of LAM-spiked urine (for human TB) and combined lactase/caseinase treatment of LAM-spiked milk (for bovine TB) enhanced 10-fold the detection levels of the LAM-test in laboratory settings.

## Results

### Improving LAM-test detection in LAM-spiked urine in the laboratory setting

First, using urine spiked with purified *M.tb* H_37_R_v_ LAM, we determined that the minimal amount of LAM that the LAM-test detects in urine is 0.0005 μg (or 500 pg, Fig. 1B). We further observed that at higher LAM concentrations (50–200 μg of LAM/ml of urine), the detection of soluble LAM by the LAM-test was worse. We also observed that the optimal recognition of LAM by the LAM-test ranged from 0.05–10 μg of LAM/ml of urine (Fig. 1B). Importantly, in repeated experiments, we found consistent results.

Seeking methods to improve the LAM-test in a POC setting, we evaluated several field feasible options; the first, urine delipidation removing inherent lipids that could interfere with the LAM-test detection, and second, urine enzymatic treatment with different hydrolytic enzymes. Our results indicate that extracting with chloroform lipids present in urine spiked with 0.001 μg (or 1 ng) of LAM, we were able to increase the band intensity detection of the LAM-test (Fig. 1C), inferring that LAM molecules were detected better. This result indicates that natural lipids present in urine interfere with the LAM-test performance.

**Figure 2:**
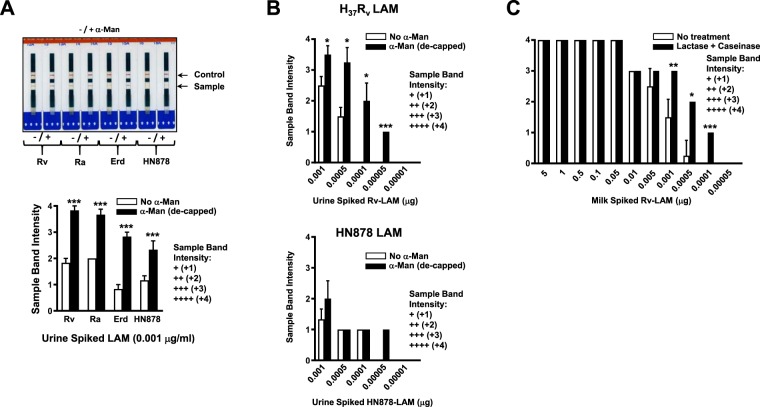
(**A**) Alere Determine LAM Ag test (LAM-test) performed in *M.tb* H_37_R_v,_ H_37_R_a_, Erdman (Erd) or HN878 LAM-spiked urine treated with α-mannosidase to remove the mannose-caps of LAM. (**B**) A quick α-mannosidase treatment step for LAM-spiked urine (from two different *M.tb* strains, H_37_R_v_ (upper graph) and HN878 (lower graph) allows the detection of this molecule in urine by the LAM-test at lower concentrations. (**C**) Lactase and caseinase treatment of LAM-spiked milk also allows the detection of this molecule in milk by the LAM-test at lower concentrations. Student’s *t* test, treatment *vs*. non-treatment, n = 3–6, using LAM-spiked urine/milk from different human/animal donors; *p < 0.05; **p < 0.005; ***p < 0.0005.

Importantly, out of all the enzymatic treatments tested, when urine spiked with 0.001 μg of LAM from different *M.tb* strains was treated with 0.1 IU of α-mannosidase at room temperature, results showed that this very simple step done before performing the LAM-test significantly increased the intensity of the LAM-test detection band (Fig. 2A**)**. This α-mannosidase treatment removes terminal 2-linked mannose residues in both, the mannose caps and from the single 2-mannose branched mannan-core of LAM (Fig. 1A). We observed this improvement in LAM-test detection in urine spiked with structurally diverse LAMs obtained from different *M.tb* strains. (Fig. 2A). Further, using *M.tb* H_37_R_v_ LAM spiked urine, α-mannosidase treatment increased LAM-test detection levels to be as low as 0.00005 μg (or 50 pg) of LAM/ml of urine, a 10-fold detection improvement when compared to the LAM-test without α-mannosidase treatment of urine, which could only detect as low as 0.0005 μg/ml of urine (Figs. 1B and 2B). Interestingly, although the LAM-test was not consistent in detecting LAM from different strains of *M.tb*, at lower levels (0.0005 μg), it was capable of detecting structurally different LAMs from different *M.tb* strains (Fig. 2B). As expected, controls consisting of α-mannosidase treatment of non-spiked LAM urine were LAM-test negative (data not shown).

### Improving LAM-test detection in LAM-spiked milk

We further assessed the efficiency of the LAM-test to detect LAM in milk, an easy specimen to obtain in the field, for the purpose of determining if the LAM-test could be used for the bovine TB diagnosis in a POC setting. Unexpectedly, no improvement was observed with LAM-spiked milk treated with α-mannosidase (data not shown). Indeed, the LAM-test was able to detect LAM in milk and detection levels were 10-fold improved (from 0.001 μg/ml to 0.0001 μg/ml) after LAM-spiked milk was treated with both caseinase and lactase (Fig. 2C).

## Discussion

Currently, there are three tests for the detection of LAM (whole or fragmented) in human samples, the LAM-test^7^, the Lionex test^17^, and the new FujiLAM test^18^. Of these, currently only the LAM-test is WHO supported for the diagnosis and screening of active TB in people living with HIV^9,19^. In order to improve the efficacy of this test in detecting LAM in urine, we accounted for the LAM structural characteristics and how urine acts as a buffer. Without further sample manipulation, the LAM-test could detect as low as 0.0005 μg of LAM/ml of urine. Interestingly, we encountered that large amounts of LAM in urine interfered with the reading in a way that the intensity of the visible band on the LAM-test strip had lower intensity at the LAM concentrations ranging from 100–200 μg/ml. This could be due to micelle formation of LAM in urine affecting the LAM epitopes disposition of being recognized by the LAM-test polyclonal antibodies. In this regard, LAM contains up to 4 fatty acids and thus, LAM micelle formations in aqueous buffers have been described^20^. Nonetheless, LAM-test higher band intensities were observed in concentrations between 50 and 0.01 μg/ml of urine. In this regard, other studies have demonstrated a semi-quantitative relationship between LAM concentration and band intensity of the LAM test^7,21–23^, and others suggested that a darker LAM band intensity relates to a greater bacillary load of *M.tb*^24–26^. No detection of LAM was observed in concentrations below 0.0005 μg/ml of urine.

A quick step involving the removal of lipids allowed us to detect LAM in urine better. These results were observed in urine spiked with virulent *M.tb* H_37_R_v_ and the attenuated *M.tb* H_37_R_a_ LAM types; however, this was not observed for the *M.tb* Erdman LAM. This indicates the importance of the LAM structure from different *M.tb* strains (reviewed in^4,27^) and their spatial conformation when dissolved in urine, as well as the interactions of LAM molecules with other lipids present in urine in determining how well the LAM-test detects this molecule.

After exposure of urine to several enzymatic treatments (Proteinase-K, non-specific esterase, phospholipase, phosphatase, urease, creatinase) failed to improve the LAM-test (data not shown), we opted to determine if the removal of the mannose-caps from LAM using α-mannosidase was able to further enhance the detection of this molecule by the LAM-test in urine. Our results indicate that a 15-minutes treatment of urine with 0.1 IU of α-mannosidase in the lab setting enhanced the recognition of LAM by the LAM-test. This result was observed for the LAMs of all *M.tb* strains tested. Thus, removal of the mannose caps of LAM may uncover specific arabinan epitopes of this molecule and probably also changes its spatial conformation, increasing the affinity of the LAM-test polyclonal antibodies to recognize and bind LAM, increasing by 10-fold the detection levels of this test.

Finally, we determined if the LAM-test could be useful to detect LAM in milk, and thus be used as a POC test in farms for the detection of Bovine TB in cattle. Bovine TB is an increased global health problem, with areas around the globe where 15–25% of human TB cases are directly related to *Mycobacterium bovis* infection through zoonotic transmission or consumption of *M. bovis* contaminated products (*i.e*. unpasteurized milk)^28^. The LAM-test could in fact detect well LAM in milk at the lowest concentration of 0.001 μg/ml of milk. This is in contrast to the LAM concentration of 0.0005 μg that this test could detect in urine, indicating that milk contains soluble components that interfere with the detection of LAM by the LAM-test. In order to improve LAM detection in milk, we tried different methods (delipidation, Proteinase-K treatment, α-mannosidase, centrifugation to analyze the plasma milk phase, etc., data not shown); however, in our hands, only the combination of lactase and caseinase treatment resulted in an improvement of LAM detection in milk, where the LAM-test was able to detect as low as 0.0001 μg/ml (or 100 pg/ml), a 10-fold improvement relative to non-enzymatically treated milk. As a caution, in milk samples and in some instances, we observed that when the LAM-test was repeated, exact band intensity readings were not repeated. However, in our LAM-test studies an initial positive reading [1+ to 4+] never gave us a negative reading [0] or vice versa when the LAM-test was repeated. This variation in band intensity from repeated tests using LAM-spiked milk samples is a concern that somehow gets mitigated with the current reading card of the LAM-test, where the appearance of a positive band at any intensity (+1 to +4) is indicative of a positive detection.

Overall these studies attempted to improve the LAM detection levels in urine/milk by the commercially available LAM-test. Two quick steps, one in urine (α-mannosidase treatment) and one in milk (lactase/caseinase-combined treatment), both for 15-minutes at room temperature, significantly reduced the concentrations of LAM in these specimens that could be detected by the LAM-test. All these enzymatic treatments can be easily performed in the field as part of the POC LAM-test, adding only 15 minutes to the 25 min that this test already requires. Importantly, from the point of view of the added cost, currently the LAM-test cost is US$3.5 per test. The addition of the α-mannosidase urine treatment will add the cost of US$0.45 to the LAM-test; and the combined addition of lactase and caseinase milk treatment will add the cost of US$0.48 per test, allowing the detection of lower concentrations of LAM by the LAM test that otherwise will be missed.

A major limitation of this study is that the α-mannosidase treatment was tested on urine and milk spiked with purified bacterial LAM, and thus the need for separate clinical research studies specifically designed to address the potential of our findings. Our current efforts are directed to test this treatment in ongoing pilot studies in different high TB burden sites (e.g. Guatemala, Ethiopia, Mozambique). It is thought that immunological properties of LAM detected in the urine of TB patients are quite different from those of the antigen directly purified from bacteria^18^, thus future field studies will determine if α-mannosidase treatment of urine allows better detection of the natural secreted form of LAM in urine from active TB patients.

These fast and easy to perform biochemical treatment could potentially also improve other POC tests, such as the novel FujiLAM, which detects the MTX present in the mannose-caps of LAM (FIND personal communication). In this instance, treatment with α-mannosidase should remove all mannose-caps from ManLAM except the one containing MTX, and thus, potentially this could be better exposed to the recognition by FujiLAM test specific MTX Abs. This hypothetical reasoning will need to be further evaluated in future studies when the FujiLAM test will be commercially available.

Further studies are required to replicate and corroborate our laboratory results in field settings with scarce diagnostic techniques and delayed diagnosis^29^ using urine from human subjects with presumable symptoms of active TB, as well as using milk from cattle presumable with symptoms of bovine TB.

## Methods

### Human subjects and ethics statement and specimens

All experiments involving human specimens (urine) were performed in accordance with relevant guidelines and regulations. For laboratory studies, pre-existing human urine samples were obtained from anonymous healthy volunteers [Human Subjects Institutional Review Board (IRB) exempted research approved by UT-Health San Antonio IRB] in Texas.

Whole milk was obtained from unidentified healthy cows from an Ohioan farm (IACUC exempt, no animal manipulations). All specimens were spiked with known amounts of in-house purified LAMs^30,31^, as indicated in figures and figure legends, from different laboratory reference *M.tb* strains (H_37_R_v_, H_37_R_a_, Erdman) and from a hypervirulent *M.tb* clinical isolate (HN878)^30^.

### Alere determine urine LAM Ag test (LAM-test)

LAM-tests were performed as indicated by the manufacturer instructions. Urine (60 μL) was applied to LAM-test strips followed by incubation at room temperature for 25 minutes. LAM-test results were then inspected by the naked-eye. In the lab setting, the intensity of any visible band on the LAM-test was graded as positive and from +1 (minimum intensity) to +4 (maximum intensity) depending on its intensity and following the post 2014 reference card (new grades from +1 to +4) provided by the manufacturer. Readings were independently performed by two to three researchers in a blinded manner following manufacturer’s instructions.

### Delipidation of urine and milk

Chloroform was added to LAM-spiked urine/milk 1:1 (v/v), hand mixed and led settle for 15 minutes at room temperature until bipartition was observed. The aqueous phase containing LAM was then directly used to perform LAM-tests following manufacturer’s instructions.

### Enzymatic treatments of urine and milk

Proteinase-K, non-specific esterase, phospholipase, phosphatase, urease, or α-mannosidase, (each at 0.1 IU, Sigma-Aldrich, Saint Louis, MO) was added to 150 μl of LAM-spiked urine, and lactase and caseinase (both at 0.1 IU, Sigma-Aldrich) were added to 150 μl of LAM-spiked milk at the concentrations indicated in the figures and figure legends, hand mixed and further incubated for 15 minutes at room temperature. Enzymatically treated LAM-spiked urine or milk was then directly used to perform LAM-tests following manufacturer’s instructions.

### Statistical analysis

Experiments were performed using urine or milk from different human or animal donors, respectively. Unpaired two-tailed Student’s *t*-test was used for two group comparisons (delipidation/enzymatic treatment *vs*. non-treatment). Statistical significance was determined using Prism 4 GraphPad software and reported as *p < 0.05; **p < 0.005; ***p < 0.0005.

## Data Availability

All materials, data and associated protocols will be promptly available to readers upon proper request.
